# Effect of Quaternary Ammonium Carboxymethylchitosan on Release Rate *In-vitro *of Aspirin Sustained-release Matrix Tablets

**Published:** 2013

**Authors:** Lingbin Meng, Zhongqiu Teng, Nannan Zheng, Weiwei Meng, Rongji Dai, Yulin Deng

**Affiliations:** a *School of Life Science, Beijing Institute of Technology, Beijing, 100081, P. R. China*; b *Sanofi-Aventis Investment Co., Ltd., Beijing, 100013, P. R. China.*^*1*^*These Authors Contributed Equally to this Work.*

**Keywords:** Chitosan derivatives, Drug release rate, Aspirin, Sustained-release matrix tablet

## Abstract

The aim of this study was to develop a derivative of chitosan as pharmaceutical excipient used in sustained-release matrix tablets of poorly soluble drugs. A water-soluble quaternary ammonium carboxymethylchitosan was synthesized by a two-step reaction with carboxymethylchitosan (CMCTS), decylalkyl dimethyl ammonium and epichlorohydrin. The elemental analysis showed that the target product with 10.27% of the maximum grafting degree was obtained. To assess the preliminary safety of this biopolymer, cell toxicity assay was employed. In order to further investigate quaternary ammonium carboxymethylchitosan application as pharmaceutical excipient, aspirin was chosen as model drug. The effect of quaternary ammonium CMCTS on aspirin release rate from sustained-release matrix tablets was examined by *in-vitro *dissolution experiments. The results showed that this biopolymer had a great potential in increasing the dissolution of poorly soluble drug. With the addition of CMCTS-CEDA, the final cumulative release rate of drug rose up to 90%. After 12 h, at the grade of 10, 20 and 50 cps, the drug release rate increased from 58.1 to 90.7%, from 64.1 to 93.9%, from 69.3 to 96.1%, respectively. At the same time, aspirin release rate from sustainedrelease model was found to be related to the amount of quaternary ammonium CMCTS employed. With the increase of CMCTS-CEDA content, the accumulated release rate increased from 69.1% to 86.7%. The mechanism of aspirin release from sustained-release matrix tablets was also preliminary studied to be Fick diffusion. These data demonstrated that the chitosan derivative has positive effect on drug release from sustained-release matrix tablets.

## Introduction

Chitosan is a liner polysaccharide derived by *N*-deacetylation of the natural biopolymer chitin, which is the second most abundant naturally occurring polymer in nature. Due to the different polymerization degree, the molecular weight of this polymer is between 50~1000 kDa. Because of its biocompatibility, biodegradability and FDA approval, this biopolymer has been widely applied in various areas such as additives in food industry, biomaterials in tissue engineering and excipients in pharmaceutical industry (1, 2). In the past few decades, chitosan and its derivatives have been more frequently employed in various routes of drug delivery (3-6). The earliest report showed that the chitosan was able to promote the transmucosal absorption of small polar molecules from nasal pithelia (7). At the same time, several studies showed that those polymers were effective and safe absorption enhancers to improve mucosal (nasal, peroral) delivery of hydrophilic macromolecules such as heparins, peptide and protein drugs (8-11). Both nasal and oral drug delivery researches have demonstrated that a lot of macromolecular drugs can be transported signiﬁcantly after co-administration with chitosan (12-15). In addition, chitosan and its derivatives have been shown to excel in trans-cellular transport. Wenguang Liu had summarized the recent encouraging advances in unveiling the mechanism of cell entry and application of chitosan derivatives as novel nonviral vectors (16). Pharmaceutical applications of those polymers contained oral, nasal, ocular, parenteral and transdermal drug delivery over recent years (2). However, more importantly, the amount of derivative groups and the physical form of chitosan have also shown to contribute to the biological properties of chitosan (17-20). In general, water-soluble drugs are more suitable for sustained-release matrix tablets. Due to poor solubility of chitosan in water, a lot of chitosan derivatives have been evaluated to overcome this deficiency. It has been found that N-trimethyl chitosan (TMC) showed excellent solubility over a wide pH range, suggesting that it can be employed as an absorption enhancer in neutral and basic environments (9). These enhancing effects were demonstrated by a decrease in the transepithelial electrical resistance (TEER) values across epithelial cell monolayers (Caco-2) as well as the increase in transport of large hydrophilic compounds across these monolayers at neutral pH values. Moreover, the quaternization degree of the biopolymer was found to play an important role for its absorption enhancing properties (19-21).

In this context, after the introduction of long chain quaternary ammonium groups, a chitosan derivative which was soluble in water was synthesized using CMCTS, decylalkyl dimethyl ammonium and epichlorohydrin. Compared with the main cosolvent currently used in sustained-release formulation, chitosan and its derivative have the advantage of being easily accessible, biologically secure and environmentfriendly. This polymer is suggested to be also biodegradable similar to chitosan because both of them have the same main body (22-23). The solubility determination experiment results showed that the solubility in water of this polymer was about 0.04 g·mL^-1^. In addition, this chitosan derivative could overcome some weakness of chitosan and possess some improved properties, such as being non-toxic and biocompatible. Generally, the sustained-release formulations of poorly soluble drugs have poor dissolution performance. Aspirin, a representative of those drugs was chosen to be a model drug. According to the characteristics of this biopolymer, the major objective of this work was to investigate chitosan derivative and its ability to increase aspirin release from sustained-release matrix tablets.

## Experimental

Ethyl cellulose **(**EC) and lactose were obtained from Beijing Fenglijingqiu Commerce and Trade Co., Ltd. Three viscosity grades of EC (10, 20 and 50 cps) were used in present work. CMCTS was purchased from Zhejiang Aoxing Biotechnology Co., Ltd. Aspirin was purchased from Shandong Ruitai Chemicals Co., Ltd. All other chemicals and regents were ACS grade.

*Synthesis of CMCTS-CEDA*

Typical procedure for the synthesis of chlorinated epoxypropyl decylalkyldimethyl ammonium was as follow (24-30). Decylalkyl dimethyl ammonium (19.7 g) and epichlorohydrin (13.0 g) were added to 250 mL round bottom flask, and reacted overnight at 25°C under continuous magnetic stirring. Ethyl acetate was used to wash the product for three times. And then, 25.3 g of chlorinated epoxypropyl decylalkyldimethyl ammonium (CEDA) was obtained after evaporation (yield = 85.4%). 

CMCTS (0.5 g) was immersed in 20 mL N-methyl pyrrolidone overnight at room temperature (RT), and after the addition of NaOH (0.4 g), the system was stirred for 2 h at 60°C. CEDA (3.0 g) and NaOH (0.1 g) was dissolved in N-methyl pyrrolidone at RT for 2 h, and this mixture was added to the above mentioned system. After 10 h of reaction at 60°C, the mixture was dissolved in a suitable amount of water and precipitated by adding proper alcohol. Finally, 3.8 g of chlorinated epoxypropyl decylalkyldimethyl ammonium carboxymethylchitosan (CMCTS-CEDA) was obtained (yield = 95%).

*Cell toxicity test*

Methyl thiazolyl tetrazolium (MTT) experiment was accepted to assess the cytotoxicity of this chitosan derivative. Firstly, the cell cultures were inoculated to 96 shadow masks and cultivated under a condition of 37°C for 24 h. After that, a series of CMCTS-CEDA gradient solutions (0.1, 1, 10 and 100 μg·mL^-1^) were added to those cell cultures, and then maintained them at 37°C for 24 h. Secondly, 20 μL of MTT (5 mg·mL^-1^) was added to those cultures, cultivated them under a condition of 37°C for 6 h. Next, the supernatants were removed and mixed residue with 150 μL dimethyl sulfoxide (DMSO). Meanwhile, a blank and a negative control experiment were also performed at the same time. The optical density (OD) values of samples were determined at 570 nm of wavelength with an ELISA.

*Tablets design and formulation*

Firstly, tablets containing 25% of drug were prepared to study the effects of CMCTSCEDA on the drug release rate. They weighed 180~200 mg each and were subjected to three viscosity grades of EC: 10, 20 and 50 cps. The rest of tablets were added with lactose. The six formulations were shown in Table 1. 

**Table 1:** Different formulations of tablets with or without CMCTS-CEDA

**Formulation**	**Aspirin** **(%, w/w)**	**Lactose** **(%, w/w)**	**EC**		**CMCTS-CEDA(%, w/w)**
			**(%, w/w)**	**Grade **(cps)	
1	25	35	40	50	0
2	25	35	40	20	0
3	25	35	40	10	0
4	25	35	40	50	10
5	25	35	40	20	10
6	25	35	40	10	10

Those tablets were compressed with a compaction force of 20 kN utilizing a ZP8-type Rotary Tablet Press (Shanghai Xinyuan Pharmaceutical Co., Ltd.). The resulting tablets containing EC, lactose, aspirin and CMCTS-CEDA were ﬂat-faced, 8.0 mm in diameter and 3.0 mm in thickness. The hardness of tablets (n = 20) was measured using a hardness tester (Tianjin Guoming Medical Equipment Co., Ltd.). Secondly, tablets containing 25% of drug and 40% of EC (50 cps) were prepared to examine the effect of CMCTSCEDA content on drug dissolution rate. Four different CMCTS-CEDA contents of tablets: 0.1%, 0.5%, 1.0% and 2.0% were investigated, respectively. The formulations were shown in Table 2. 

**Table 2:** Formulation with different contents of CMCTS-CEDA in aspirin tablets

**Formulation**	**Aspirin (%)** **(%, w/w)**	**Lactose (%)** **(%, w/w)**	**EC (%)** **(%, w/w)**	**CMCTS-CEDA (%)** **(%, w/w)**
7	25	34.9	40	0.1
8	25	34.5	40	0.5
9	25	34.0	40	1.0
10	25	33.0	40	2.0

Finally, the dissolution enhancement by addition of CMCTS-CEDA was compared with that by the addition of CMCTS. The tablets contained 25% of aspirin, 34% of lactose and 40% of EC (50 cps). The remaining parts of tablets were 1% of CMCTS-CEDA and CMCTS, respectively. The formulations were shown in Table 3.

**Table 3:** Formulation with 1.0% of CMCTS-CEDA and CMCTS in aspirin tablets

**Formulation**	**25% (w/w)**	**34% (w/w)**	**40% (w/w)**	**1% (w/w)**
11	Aspirin	Lactose	EC (50 cps)	CMCTS-CEDA
12	Aspirin	Lactose	EC (50 cps)	CMCTS

*Drug release evaluation*

According to the provisions of the Chinese Pharmacopoeia 2010 about sustained release tablets, the *in-vitro *dissolution test was performed using a dissolution apparatus (RCZ- 8B, Tianjin Tiandatianfa Technology Co., Ltd.). Those tablets were individually placed into 500 mL dissolution medium (0.1 M HCl) in an apparatus with a rotating paddle (100 r.p.m). The beakers were placed into circulating water bath and incubated at 37 ± 0.5°C throughout the whole release study. To prevent the tablets from sticking to the glassware, a small and curved grid was placed at the bottom of the recipient to allow the release of drug from all sides of the matrix. 10 mL of sample was collected automatically at 0.5, 1, 2, 4, 6, 8, 10 and 12 h respectively and replaced with an equal volume of fresh dissolution medium. All samples were filtered through a 0.45 μm filter before detection. The samples were assayed at 275.6 nm of wavelength via UV spectrophotometer (TC-181, Beijing Persee General Instrument Co., Ltd.). For each formulation, at least 12 dissolution runs were carried out and the averaged results are reported below. The release time can be obtained from the *x*-axis, while the cumulative of release rate was depicted on the *y*-axis.

 Comparison of release profiles was investigated by a similarity factor which can be defined as (31-32): 


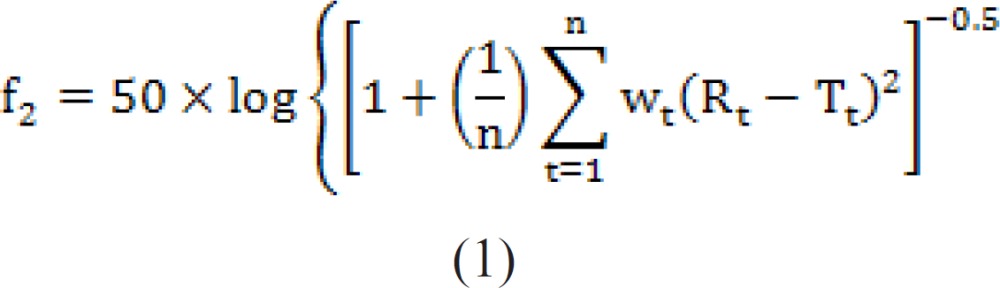


In the equation above, f_2_ is the similarity factor, n is the number of time point, R_t_ is the mean percent drug dissolved of *e.g*. the current formulation, and T_t_ is the mean percent drug dissolved of *e.g*. the changed composition. An f_2_ value between 50 and 100 suggests that the two dissolution proﬁles are similar. 

Furthermore, the mechanism of drug release from sustained-release matrix tablets was studied preliminary by ﬁtting the dissolution profiles to the following equations: zero-order, ﬁrst-order, Higuchi equation and Ritger-Peppas model, respectively (33-34).

*Formulation design and optimization*

Orthogonal design was employed to optimize the formulation (35). On the basis of single factor evaluation, three variables were fixed as follows: the content of EC, the viscosity grade of EC and the quantity of CMCTS-CEDA. L_9_ (34) of orthogonal table was designed to prepare the sustained-release matrix tablets. The drug release degree of tablets at 2, 6 and 8 h were explored according to the provisions of the Chinese pharmacopoeia 2010. The cumulative release rate at 2, 6 and 8 h was more than 30%, 50% and 75%, respectively. This was the foundation of formulation selecting. The variables and their ranges are summarized in Table 4. 

**Table 4:** Factors and levels of formulation variables in orthogonal design

**Factor**	**Level**		
	**1**	2	3
A (EC, %, w/w)	30.0	40.0	50.0
B (EC viscosity, cps)	10.0	20.0	50.0
C (CMCTS-CEDA, %, w/w)	0.5	1.0	2.0

The high and low values of each variable were defined according to the preliminary experiments. Experimental design requires specific evaluation indicators to assess whether the test data was perfect or not. According to the requirements about sustained-release tablet of the Chinese pharmacopoeia 2010, the comprehensive grading method was chosen to evaluate the results of orthogonal design in this paper. The value of K (colligation score) was defined as:

K=│Q_2_- 30%│+│Q_6_- 50%│+│Q_8_- 75%│                     (2)

where Q_2_, Q_6_ and Q_8_ were the cumulative release rate at 2, 6 and 8 h, respectively. Correlation analysis and ANOVA were employed to analyze the results of orthogonal experiment. The lower the value of K, the closer the release rate was to the standard selection. That means the formulation was better.

*Statistical and pharmacokinetic data analysis*

Data obtained were subjected to correlation analysis and ANOVA. Dissolution proﬁles were compared using similarity factor, f_2_, and the proﬁles were signiﬁcantly different if f_2_ < 50. Drug-release proﬁle reproducibility was excellent as the SD values observed for the % of drug released versus time were generally lower than 3.0%, ranging from 1.0 to 3.0%.

## Results and Discussion

*The synthesis and grafting degree calculation of CMCTS-CEDA*

**Figure 1:**
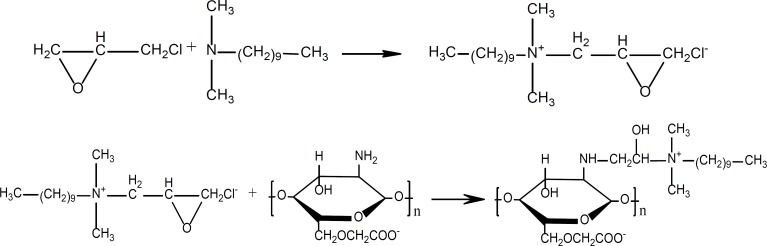
Synthesis steps of CMCTS-CEDA

CMCTS, decylalkyl dimethyl ammonium and epichlorohydrin were to synthesize CMCTSCEDA by the grafting reaction under the alkaline condition. The reaction steps are shown in Figure 1. 

The results of elemental analysis with CMCTS and CMCTS-CEDA are shown in Table 5. Since the target product didn’t have any other small molecules, the grafting degree could be calculated according to the ratio of n_C_ and n_H_. The grafting degree was calculated according to the equation as follows:

$n\mathrm{CMCTS}\times \frac{1}{x+1}n\mathrm{CEDA}\frac{x}{x+1}=n_{c-o}$                    (3)

Where x is the grafting degree, n_CMCTS_, n_CEDA_ and n_C-O_ are the ratio of n_C _and n_H_ in CMCTS, epoxypropyl decyl dimethyl ammonium chloride and CMCTS-CEDA, respectively. According to the elemental analysis and scientific calculation performed, the final product with 10.27% of the maximum grafting degree was obtained after process optimized.

**Table 5:** The content of organic elements in starting material and product

**Elements**	**CMCTS %**	**CMCTS-CEDA %**
C	28.63	30.09
H	4.21	5.22
N	2.35	4.58

*Biocompatible evaluation*

The relative growth rate (RGR) of the cell was calculated by the equation as follows. 

$\mathrm{RGR}=\frac{A_{570}-A_{570}^{0}}{A_{570}^{'}-A_{570}^{0}}$                     (4)

A_570_-- Absorbance of the experimental group, 

A^0^_570_-- Absorbance of the blank control experimental group,

A′_570_-- Absorbance of the negative control experimental group.

The relative growth rate (RGR) was used to evaluate the biocompatible evaluation. Absorbance value of samples and results of biocompatible evaluation were illustrated in Table 6. It was easy to obtain the RGR of the cell after adding different concentration test solution to the cell culture. The RGR of the cell was between 93.43% and 101.23%. Those results showed that the biological material for cell was non-toxic.

**Table 6:** Absorbance value of samples at wavelength of 570 nm

**Sample**	**Negative control**	**CMCTS-CEDA solutions (μg/mL)**			
		**0.1**	**1**	**10**	**100**
1	1.332	1.349	1.608	1.484	1.392
2	1.597	1.583	1.426	1.335	1.362
3	1.536	1.486	1.351	1.489	1.442
4	1.443	1.507	1.455	1.424	1.328
5	1.398	1.471	1.383	1.406	1.302
General average	1.461	1.479	1.445	1.428	1.365
Blank control	0.086	0.083	0.067	0.075	0.087
RGR	1.000	1.012	0.9886	0.977	0.934

*The evaluation of CMCTS-CEDA on drug release rate*

*Effect of CMCTS-CEDA addition on drug release*

The similarity factors among different formulations with three viscosity grades of EC are shown in Table 7. 

**Table 7:** The similarity factors of different formulations

**Formulation**	**Formulation**	**f**_2_
1	2	59.6
1	3	44.5
2	3	58.7
4	5	99.9
4	6	99.8
5	6	99.9

The effect of CMCTSCEDA on aspirin release profiles form sustained release matrix tablets are depicted in Figure 1. After the addition of CMCTS-CEDA, an increase in the release rate of aspirin was observed. Those results of experiment showed that the addition of CMCTS-CEDA could significantly improve the dissolution of the drug. Moreover, the final cumulative release rate of drug rose up to 90% in spite of any grade of EC. After 12 h, at the grade of 10, 20 and 50 cps, the drug release rate increased from 58.1 to 90.7%, from 64.1 to 93.9%, from 69.3 to 96.1%, respectively. We could conclude that CMCTS-CEDA had an active influence on aspirin release from the sustained-release matrix tablets. 

In addition, with the increase of EC viscosity, the release rate of aspirin from tablets became slower independently from CMCTS-CEDA. When the hardness of the tablets was fixed, the low viscosity of EC was more easily compressed than the high viscosity. As a result, the drug release rate of the low viscosity was faster than the high viscosity of EC.

*Effect of CMCTS-CEDA contents on drug release*

The similarity factors among formulations with different content of CMCTS-CEDA were summarized in Table 8.

**Table 8:** The similarity factor of formulations with different content of CMCTS-CEDA

**Formulation**	**Formulation**	**f**_2_
7	8	49.5
7	9	43.1
7	10	37.2
8	9	68.8
8	10	53.5
9	10	66.8

**Figure 2:**
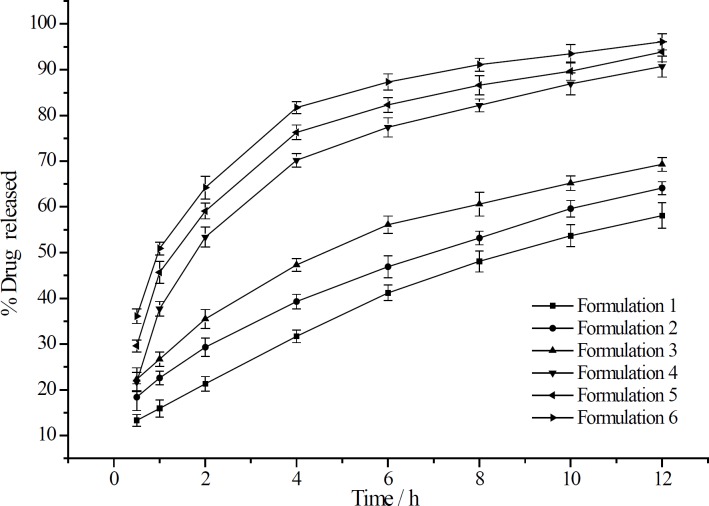
The release curve of aspirin from sustained-release matrix tablets containing three grades of EC with or without CMCTS-CEDA (mean ± SD, n = 12).

 The effect of different CMCTS-CEDA contents on the release rate from sustained release matrix tablet was also studied. Four levels of CMCTS-CEDA (0.1%, 0.5%, 1.0% and 2.0%) were chosen to investigate the effect of this biopolymer on aspirin delivery. Based on the four different contents of CMCTSCEDA, the release profiles were showed in Figure 2.

 After 12 h, with the increasing of CMCTSCEDA content, the accumulated release rate increased from 69.1% to 86.7%. Those profiles indicated that the content of CMCTS-CEDA in those formulations had significant impact on drug delivery rate from sustained release matrix tablet. The release rate curves of drug accumulation showed that an amount of drug released from tablets significantly improved as the CMCTS-CEDA content increased. Because the polymer is water-soluble, it could promote the disintegration of the matrix. With the increase of the amount of polymer, the disintegration rate of this matrix also increased. As a result, more drug was released from the tablets. 

*Effect of CMCTS-CEDA and CMCTS on* *drug release*

**Figure 3:**
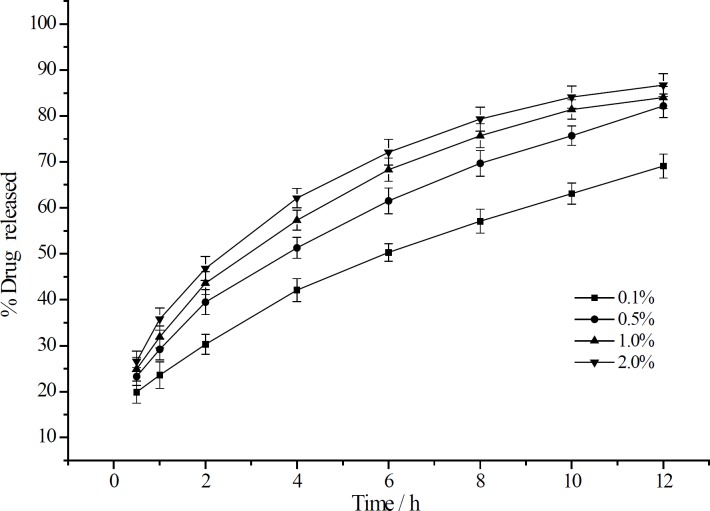
The release curve of aspirin from sustained-release matrix tablets with different CMCTS-CEDA contents (mean ± SD, n = 12).

The dissolution enhancement by the addition of CMCTS-CEDA was compared with that by the addition of CMCTS as shown in Figure 3. Compared to the addition of CMCTS, CMCTSCEDA could significantly increase the aspirin release rate from sustained-release matrix tablets. The release rate of tablet with CMCTSCEDA at 12 h was 31.7 % more than CMCTS ones (CMCTS: 52.3%, CMCTS-CEDA: 84.0%).

**Figure 4:**
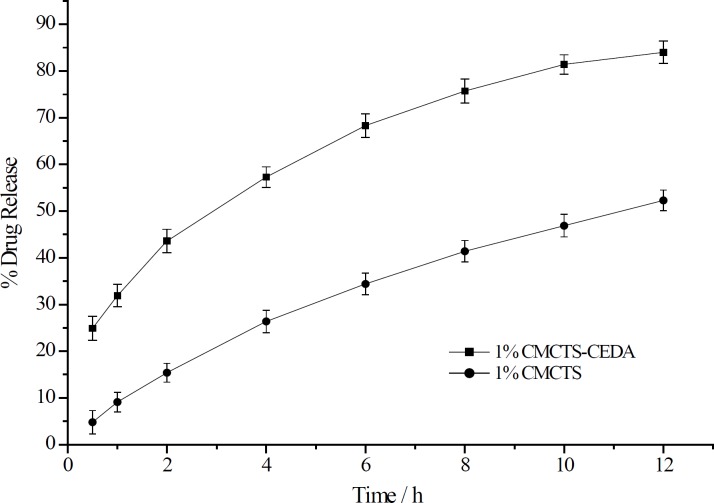
The release curve of aspirin from sustained-release matrix tablets with addition of CMCTS-CEDA and CMCTS (mean ± SD, n = 12).

*The optimization of aspirin formulation*

According to the L_9_ (3^4^) orthogonal formulations were obtained. Besides, the formulation design and drug release rate were summarized in Table 9. 

**Table 9:** Formulation design and results

**No.**	**A** **(%, w/w)**	**B** **(cps)**	**C** **(%, w/w)**	**% Drug Release**		
				**2h**	**6h**	**8h**
1	30	10	0.5	62.5	70.4	74.3
2	30	20	1.0	65.3	66.2	68.4
3	30	50	2.0	70.2	76.1	78.2
4	40	10	1.0	53.7	60.2	61.7
5	40	20	2.0	59.8	64.6	67.1
6	40	50	0.5	59.6	61.8	65.4
7	50	10	2.0	53.1	64.6	68.3
8	50	20	0.5	46.1	55.5	58.8
9	50	50	1.0	53.9	59.2	60.7

After using the dissolution tests above mentioned, all tablets were measured. Correlation analysis and ANOVA of orthogonal processing data are shown in Table 10 and Table 11, respectively. According to the data analysis value of range shown in Table 10 and the value of F in Table 11, the sequence of various factors’ effects on index order could be drawn as: A > B > C. And according to the value of K in Table 10, the best level of each factor was followed as: A: 3 > 2 >1, B: 1 > 2 > 3 and C: 1 > 2 > 3. Hence, the best combination of the three factors was A_3_B_1_C_1_, corresponding to the following formulation: the content of EC, 50%, the viscosity of EC, 10 cps and the content of CMCTS-CEDA, 0.5%. 

According to the release test results of aspirin optimal formulation, preliminary release mechanism of aspirin sustained-release matrix tablets was studied. Four regression equations which fitted the percentage of the accumulated drug dissolution (M_t_/M_∞_) on release time (t) were acquired as followed:

y = 0.0567x + 0.2488 (r= 0.9543)                    (5)

y = -0.2124Inx + 0.7098 (r = 0.9668)                     (6)

y = 0.2437x^0.5^ + 0.0383 (n = 0.5,r = 0.9982)                    (7)

y = 0.2784x^0.4493^(n = 0.4493,r = 0.9983)                     (8)

The four equations were corresponding to the four models: zero-order, ﬁrst-order, Higuchi equation and the Ritger-Peppas model, respectively. The value of correlation coefficient (r) in Equation (7) and Equation (8) were more than 0.998, and the value of n in Equation (8) was less than 0.45. These results illustrated that the Higuchi equation and the Ritger-Peppas model can be fitted well for the release process of this sustained-release matrix tablets of aspirin. It can be preliminarily concluded that aspirin is released from sustained-release matrix tablets in the form of Fick diffusion mechanism. The progress depicted that the drug was dissolved in PBS through the matrix, and then spread from the matrix.

**Tabel 10:** The correlation analysis of orthogonal processing data

**No.**	**EC** **Content (%, w/w)**	**EC** **Viscosity (cps)**	**CMCTS-CEDA** **Content (%, w/w)**	**K**
1	1	1	1	56.9
2	1	2	2	59.9
3	1	3	3	74.8
4	2	1	2	48.0
5	2	2	3	54.3
6	2	3	1	54.5
7	3	1	3	44.4
8	3	2	1	37.7
9	3	3	2	47.4
Mean 1	63.9	49.8	49.7	
Mean 2	52.3	50.6	51.8	
Mean 3	43.2	58.9	57.8	
Range	20.7	9.1	8.1	

**Table 11:** The variance analysis of orthogonal processing data

**Indicators**	**Factor**	**Squares of deviations**	**F**	**Significance**
Drug Release %	EC Content	645.86	888.39	*
	EC Viscosity	152.51	209.78	*
	CMCTS-CEDA Content	107.23	147.49	*

## Conclusion

In this paper, a chitosan derivative (CMCTSCEDA) was synthesized by a two-step reaction. The characterization results showed the wanted structure was obtained. Cell toxicity test showed that the biological material for cell was nontoxic. In order to study the use of this biopolymer as pharmaceutical excipient in oral delivery system, we prepared some aspirin sustainedrelease matrix tablets. The enhancing effect was demonstrated via *in-vitro *dissolution tests. After CMCTS-CEDA was added, it could significantly increase the dissolution of drug, and the final delivery rate of drug rose up to 90%. Otherwise, it would be lower than 70%. In addition, when the content of CMCTS-CEDA was increased, the release rate of aspirin also increased. After optimization of the aspirin formulation, the best content of CMCTS-CEDA in aspirin sustainedrelease matrix tablets was 0.5%. According to the preliminary study, the drug release mechanism was Fick diffusion and we got the best regression equation (y = 0.2784 x^0.4493^, n = 0.4493, r = 0.9983). On the basis of these results we could conclude that CMCTS-CEDA has potential to enhance the release rate of drug from sustained-release matrix tablet. However, further studies should be conducted to develop this new pharmaceutical excipient.

## References

[1] Kean T, Thanou M (2010). Biodegradation, biodistribution and toxicity of chitosan.

[2] Illum L (1998). Chitosan and its use as a pharmaceutical excipient. Pharm. Res.

[3] Ramanathanand S, Block LH (2001). The use of chitosan gels as matrices for electrically-modulated drug delivery. J. Controlled Release.

[4] Hejaziand R, Amiji M (2003). Chitosan-based gastriointestinal delivery systems. J. ControlledRelease.

[5] Mao SR, Sun W, Kissel T (2010). Chitosan-based formulations for delivery of DNA and siRNA. Adv.Drug Deliver. Rev.

[6] Bhattarai N, Gunn J, Zhang M (2010). Chitosan-based hydrogels for controlled, localized drug delivery. Adv.Drug Deliver. Rev.

[7] Illum L, Jabbal-Gill I, Hinchcliffe M, Fisher AN, Davis SS (2001). Chitosan as a novel nasal delivery system for vaccines. Adv. Drug Deliver. Rev.

[8] van der Lubben IM, Verhoef JC, Borchard G, Junginger HE (2001). Chitosan and its derivatives in mucosal drug and vaccine delivery. Eur. J. Pharm. Sci.

[9] van der Merwe SM, Verhoef JC, Verheijden JHM, Kotzé AF, Junginger HE (2004). Trimethylated chitosan as polymeric absorption enhancer for improved peroral delivery of peptide drugs. Eur. J. Pharm. Biopharm.

[10] Georgeand M, Abraham TE (2006). Polyionic hydrocolloids for the intestinal delivery of protein drugs: Alginate and chitosan-a review. J. ControlledRelease.

[11] Palmberger TF, Hombach J, Bernkop-Schnürch A (2008). Thiolated chitosan-Development and in-vitro evaluation of an oral delivery system for acyclovir. Int. J. Pharm.

[12] Thanou M, Verhoef JC, Junginger HE (2001). Oral drug absorption enhancement by chitosan and its derivatives. Adv. Drug Deliver. Rev.

[13] Artursson P, Lindmark T Davis SS, Illum L (1994). Effect of chitosan on the permeability of monolayers of intestinal epithelial cells (Caco-2). Pharm. Res.

[14] Illum L, Farraj NF, Davis SS (1994). Chitosan as a novel nasal delivery system for peptide drugs. Pharm. Res.

[15] Lueßen HL, Rentel CO, Kotzé AF, Lehr CM, De Boer AG, Verhoef JC, Junginger HE (1997). Mucoadhesive polymers in peroral peptide drug delivery. IV. Polycarbophil and chitosan are potent enhancers of peptide transport across intestinal mucosae in-vitro. J.Controlled. Release.

[16] Liu WG, Kang DY (2002). Chitosan and its derivatives-a promising non-viral vector for gene transfection. J.Controlled. Release..

[17] Mi FL, Peng CK, Huang MF, Lo SH, Yang FL (2005). Preparation and characterization of N-acetylchitosan, N-propionylchitosan and N-butyrylchitosanmicros pheres for controlled release of 6-mercaptourine. Carbohydr. Polym.

[18] Lee KY, Ha WS, Park WH (1995). Blood compatibility and biodegradability of partially N-acetylated chitosan derivatives. Biomater.

[19] Schipper NGM, Varum KM, Artursson P (1996). Chitosans as absorption enhancers for poorly absorbable drugs. 1: Inﬂuence of molecular weight and degree of acetylation on drug transport across human intestinal epithelial (Caco-2) cells. Pharm. Res.

[20] Schipper NGM, Olsson S, Hoogstraate JA, DeBoer AG, Varum KM, Artursson P (1997). Chitosans as absorption enhancers for poorly absorbable drugs 2: Mechanism of absorption enhancement. Pharm. Res.

[21] Thanou MM, Kotze AF, Scharringhausen T, Lueßen HL, de Boer AG, Verhoef JC, Junginger HE (2000). Effect of degree of quaternization of N-trimethylchitosan Chloride for enhanced transport of hydrophilic compounds across intestinal Caco-2 cell monolayers. J. Controlled Release.

[22] Harish Prashanth KV, Tharanathan RN (2007). Chitin/ chitosan:Modifications and their unlimited application potentialan overview. Trends Food Sci. Tech.

[23] Rinaudo M (2006). Chitin and chitosan: Properties and applications. Prog. Polym. Sci.

[24] Sun JH, Zheng NN, Dai RJ (2009). Synthesis and Characterization of A Novel Natural Macromolecule Antibacterial Material. 3rd International Conference on Bioinformatics and Biomedical Engineering; Jun 11- 13.

[25] Meng WW, Tang HL, Chen Y, Tan HM, Wang GL (2008). A Class of Carboxymethyl Chitosan QuaternaryAmmonium Salt Derivatives and PreparationMethod. State Intellectual Property of P. R. C. Patent CN101235099.

[26] Zambito Y, Zaino C, Uccello-Barretta G, Balzano F, DiColo G (2008). Improved synthesis of quaternary ammonium-chitosan conjugates (N+-Ch) for enhanced intestinal drug permeation. Eur. J. Pharm. Sci..

[27] Thanou M, Henderson S, Kydonieus A, Elson C (2007). N-sulfonato-N, O-carboxymethyl chitosan-A novel polymeric absorption enhancer for the oral delivery of macromolecules. J. Controlled Release..

[28] Sabaa MW, Mohamed NA, Mohamed RR, Khalil NM, Abd El Latif SM (2010). Synthesis, characterization and antimicrobial activity of poly (N-vinyl imidazole) grafted carboxymethyl chitosan. Carbohydr. Polym..

[29] Zhang C, Ping QN, Zhang HJ, Shen J (2003). Synthesis and characterization of water-soluble O-succinylchitosan. Eur. Polym. J..

[30] Ma L, Li GH, Li LM, Liu P (2010). Synthesis and characterization of diethoxy phosphoryl chitosan. Int.J. Biol. Macromol.

[31] The European Agency for the Evaluation of Medicinal Products (1999). Human Medicines Evaluation (EMEA): Note for Guidance on Quality of Modiﬁed Release Products.

[32] Gil EC, Colarte AI, Bataille B, Pedraz JL, Rodríguez F, Heinämäki J (2006). Development and optimization of a novel sustained-release dextran tablet formulation for propranolol hydrochloride. Int. J. Pharm.

[33] Siepmannand J, Peppas NA (2001). Modeling of drug release from delivery systems based on hydroxypropyl methylcellulose (HPMC). Adv. Drug Deliver. Rev.

[34] Lindnerand WD, Lippold BC (1995). Drug Release From Hydrocolloid Embeddings with High or Low Susceptibility to Hydrodynamic Stress. Pharm. Res.

[35] Cheng B, Liu Q, Xiong NS (2005). The optimization of the formulation of valaciclovir sustained-release tablets by orthogonal test method. Chinese J. HospitalPharmacy.

